# A high-fat diet induces rapid changes in the mouse hypothalamic proteome

**DOI:** 10.1186/s12986-019-0352-9

**Published:** 2019-04-29

**Authors:** Fiona H. McLean, Fiona M. Campbell, Rosamund F. Langston, Domenico Sergi, Cibell Resch, Christine Grant, Amanda C. Morris, Claus D. Mayer, Lynda M. Williams

**Affiliations:** 1 Rowett Institute, University of Aberdeen Foresterhill Campus, Aberdeen, AB25 2ZD UK; 2 Division of Neuroscience, University of Dundee, Ninewells Hospital & Medical School, Dundee, DD1 9SY UK; 3 Nutrition & Health Substantiation Group, Nutrition and Health Program, Health and Biosecurity, Commonwealth Scientific and Industrial Research Organisation (CSIRO), Adelaide, SA 5000 Australia; 4 Biomathematics and Statistics Scotland, University of Aberdeen, Aberdeen, AB25 2ZD UK

**Keywords:** Hypothalamus, Proteomics, Mice, High-fat diet, Neuronal plasticity

## Abstract

**Background:**

Prolonged over-consumption of a high-fat diet (HFD) commonly leads to obesity and insulin resistance. However, even 3 days of HFD consumption has been linked to inflammation within the key homeostatic brain region, the hypothalamus.

**Methods:**

Mice were fed either a low-fat diet (LFD) or HFD containing 10% or 60% (Kcal) respectively from fat for 3 days. Mice were weighed, food intake measured and glucose tolerance calculated using intraperitoneal glucose tolerance tests (IPGTT). Proteomic analysis was carried out to determine if hypothalamic proteins were changed by a HFD. The direct effects of dietary fatty acids on mitochondrial morphology and on one of the proteins most changed by a HFD, dihydropyrimidinase-related protein 2 (DRP-2) a microtubule-associated protein which regulates microtubule dynamics, were also tested in mHypoE-N42 (N42) neuronal cells challenged with palmitic acid (PA) and oleic acid (OA).

**Results:**

Mice on the HFD, as expected, showed increased adiposity and glucose intolerance. Hypothalamic proteomic analysis revealed changes in 104 spots after 3 days on HFD, which, when identified by LC/MS/MS, were found to represent 78 proteins mainly associated with cytoskeleton and synaptic plasticity, stress response, glucose metabolism and mitochondrial function. Over half of the changed proteins have also been reported to be changed in neurodegenerative conditions such as Alzheimer’s disease. Also,in N42 neurons mitochondrial morphology and DRP-2 levels were altered by PA but not by OA.

**Conclusion:**

These results demonstrate that within 3 days, there is a relatively large effect of HFD on the hypothalamic proteome indicative of cellular stress, altered synaptic plasticity and mitochondrial function, but not inflammation. Changes in N42 cells show an effect of PA but not OA on DRP-2 and on mitochondrial morphology indicating that long-chain saturated fatty acids damage neuronal function.

**Electronic supplementary material:**

The online version of this article (10.1186/s12986-019-0352-9) contains supplementary material, which is available to authorized users.

## Background

Obesity is increasing not only in Western societies but also in the developing world [1, 2], with obesity related comorbidities and decreased life expectancy putting enormous pressure on health care systems [1, 3, 4]. The overconsumption of energy dense foods, particularly those high in saturated fat and refined sugar appears to be a primary driving force behind the obesity epidemic [5], making it important to understand the mechanisms linking diet with obesity to enable more effective preventative measures to be put in place. Dietary interactions with the hypothalamus appear to be key in the development of obesity. Thus, the aim of this study is to investigate how short-term exposure to a high-fat diet (HFD) influences the proteome of the hypothalamus to better understand how diet interacts with this brain region.

Energy balance is effectively regulated by a well-defined and complex hypothalamic system with leptin acting together with insulin in the hypothalamus not only to inhibit feeding but also to maintain peripheral glucose homeostasis [6, 7]. Nonetheless, in obesity the hypothalamus becomes insensitive to leptin and insulin signifying dysregulation of hypothalamic energy balance. A number of studies in rodents on a HFD have shown inflammation in the hypothalamus, activating both microglia and astrocytes, via the toll like receptor 4 (TLR4) [8, 9] and IKKB/NFκB inflammatory pathway, resulting in leptin and insulin insensitivity [10, 11]. The importance of this pathway is underlined by the fact that blocking or inhibiting diet-induced hypothalamic inflammation prevents leptin insensitivity, glucose intolerance and obesity [10, 12, 13]. More recently, however, the role of TLR4 in this process has been called into question [14]. HFD-induced mitochondrial dysfunction may be the origin of hypothalamic dysfunction and inflammation with excessive dietary intake leading to mitochondrial overload and oxidative stress activating the NFκB inflammatory pathway [15].

Data has emerged which indicates that microglial proliferation is only seen after 8 weeks on HFD despite increased pro-inflammatory gene expression after just 3 days [16]. This indicates that the primary inflammatory response is independent of hypothalamic immune cells with neurons being implicated in triggering microgliosis via advanced glycation end products (AGEs) [17]. In order to explore the initial mechanisms linking a HFD to hypothalamic dysfunction we have used a proteomics approach to identify key proteins changed after just 3 days of a HFD and extended these findings to N42 cells challenged with PA and OA, representative dietary long-chain saturated and monounsaturated fatty acids respectively, and present in large amounts in the HFD, to look at protein changes together with the shape and area occupied by mitochondria indicative of mitochondrial functionality.

## Methods

### Animals

Male C57Bl/6 J mice, 10 weeks of age (Harlan, Bicester, UK), were first habituated to single housing on grid floors for 1 week then changed from chow and habituated to a semi-purified low-fat diet (LFD) for a further week (10% of energy from fat and 3.8 kcal/g) to avoid any inappetence that may arise in changing directly from chow to a semi-purified diet. Single housing and grid floors were utilised to enable accurate measurement of food intake by weighing unconsumed food, to prevent coprophagia and stress due to dominance. Animals were then randomised into two groups. One group remained on the LFD while the other group were fed a HFD (60% of energy from fat and 5.2 kcal/g) ad libitum for 3 days (D12492 and D12450B, respectively; Research Diets, NJ, US) (see https://researchdiets.com/opensource-diets/dio-series-diets for complete diet composition details and Additional file 1: Table S1 for overview). The difference in fat content is due to increasing the quantity of lard in the diet. Semi–purified diets in comparison to chows have defined ingredients allowing precise diet composition facilitating the replication of experimental conditions. Food intakes and body weights were measured daily (*n* = 14 per diet). Water was supplied ad libitum but intake was not measured. Mice were killed by exsanguination under terminal anaesthesia. The brains were removed and snap-frozen over dry ice and stored at − 80 °C until proteomic studies were carried out.

### Glucose tolerance

Intraperitoneal glucose tolerance tests (IPGTTs) were carried out (*n* = 8) as a non-recovery procedure after fasting for 5 h. A blood sample (0 mins) was taken prior to an intraperitoneal (IP) glucose injection (1.5 mg/g body weight). Subsequent blood samples were taken from the tail vein at 15, 30, 60 and 120 mins and measured using an Accu Chek Aviva blood glucose monitor (Roche Diagnostics, Burgess Hill, UK). Area under the curve (AUC) was calculated using the trapezoid rule [18]. Data from this group of mice was also used as in a parallel study [19] in accordance with reducing the number of experimental animals (http://www.understandinganimalresearch.org.uk/animals/three-rs/).

### Two-dimensional gel electrophoresis (2-DE)

The hypothalamus was dissected from frozen brains (*n* = 6) using the start of the optic chiasma and the end of the median eminence as the anterior and posterior markers for the first cuts through the brain. The outer edges of the hypothalamus were then used as markers for the side cuts and the top of the third ventricle as a marker for the top of the hypothalamus for the final cut. Hypothalamic tissue was homogenised at a 1:4 ratio of tissue to buffer in 40 mM Tris pH 7.4, 0.1% *v*/v Triton X-100 containing Roche complete protease inhibitors (Sigma-Aldrich, UK) at the manufacturers recommended concentration. 2-DE was performed essentially as detailed previously with some modifications [20]. Bio-Rad, 17 cm, immobilized pH gradient (IPG) strips (pH 3–10) were used for the separation of proteins in the first dimension. Strips were rehydrated in rehydration buffer (7 M urea; 2 M thiourea; 4% *w*/*v* CHAPS; 2% w/v Biolyte; and 50 mM DTT) containing 200 μg of protein sample in a Bio-Rad IEF cell and then focused.

After the first dimension IPG strips were incubated in fresh equilibration buffer (6 M urea; 2% w/v SDS; 0.375 M Tris-HCl, pH 8.8; 20% *v*/v glycerol; and 130 mM DTT) for 10–15 min at room temperature before transfer to a second equilibration buffer (6 M urea; 2% w/v SDS; 0.375 M Tris-HCl, pH 8.8; 20% v/v glycerol; and 135 mM iodoacetamide) for 10–15 min at room temperature. The strip was then applied to the top of an 18 × 18 cm gel cassette and 5 μl of All Blue Precision Protein Standards (Bio-Rad) was loaded in the reference well. Gels were run at 200 V for 9.5 h or until the bromophenol blue had reached the bottom of the gel. After the second dimension run, the gels were fixed and stained with Coomassie Blue. Twelve gels were run in total each gel representing an individual animal from each treatment group HFD (*n* = 6) and LFD (*n* = 6).

### Identification of mouse hypothalamic proteins

2-DE gels were analysed using Progenisis Samespots software (Nonlinear Dynamics Ltd., UK). Spots which showed differences in normalised average volume with *P* < 0.05 by ANOVA in HFD vs. LFD were cut from SDS-PAGE gels. Gel plugs were trypsinized using the MassPrep Station (Waters, Micromass, UK) protocol. Spot identification was carried out using ‘Ultimate’ nanoLC system (LC Packings, UK) and a Q-Trap (Applied Biosystems/MDS Sciex, UK) triple quadrupole mass spectrometer fitted with a nanospray ion source as described previously [20]. The total ion current (TIC) data were submitted for database searching using the MASCOT search engine (Matrix Science, UK) using the MSDB database.

### Cell culture and reagents

The N42 clonal hypothalamic neuronal cell line (mHypoE-N42) (Cellution Biosystems Inc. Burlington, Canada) was cultured in Dulbecco’s modified Eagle’s medium (DMEM) (Invitrogen Life Technologies, Paisley, UK), supplemented with 10% fetal bovine serum (FBS) and 1% penicillin/streptomycin (Invitrogen Life Technologies, Paisley, UK) and maintained at 37^o^ C under a 5% CO_2_ atmosphere. This cell line is derived from mouse hypothalamic primary cultures by retroviral transfer of SV40 T-Ag and expresses enzymatic markers, receptors and neuropeptides which makes it a valuable tool to study hypothalamic metabolic pathways [21]. Information regarding genes expressed in this cell line can be found at (https://www.cedarlanelabs.com/Products/Detail/CLU122?lob=Cellutions). The fatty acids PA and OA and fatty acid free bovine serum albumin (BSA) were from Sigma Aldrich (St. Louis, MO, USA).

### Fatty acid-BSA conjugation

PA and OA were conjugated to fatty acid free BSA as described previously [22, 23] with some modifications detailed below. PA and OA were dissolved in 0.1 M NaOH in a shaking water bath and solubilised at 70^o^ C or 37^o^ C respectively in order to yield a final concentration of 20 mM. A 0.5 mM fatty acid free BSA solution was prepared by dissolving BSA in deionised water at 55^o^ C then mixing with PA and OA in order to obtain a 1:4 BSA to fatty acid molar ratio (0.5 mM BSA, 2 mM fatty acid). The PA- and OA-BSA mixtures were vortexed for 10 s followed by 10 min incubation at 55 ^o^ C or 37^o^ C respectively before being cooled to room temperature and sterilised by passing through a 0.22 μm pore size membrane filter and stored at -20^o^ C until use.

### Western blotting

Lysates were prepared from N42 neurons after a 6 h incubation with 50 μM BSA, 200 μM OA or 200 μM PA in serum-free medium. This concentration of fatty acid was chosen as it does not cause toxicity up to 24 h of treatment in a neuronal cell line and falls within the range of systemic concentrations of free fatty acids reported [22, 24].

Cells were then scraped into phosphate-buffered saline (PBS) and pelleted by centrifugation, M-PER mammalian protein extraction reagent (Thermo scientific) was added before sonication using a Sanyo Soniprep 1500 to ensure complete cell lysis. Protein concentrations were determined using the Pierce 660 nm protein assay reagent (Thermo Scientific). After addition of 4X Laemmli sample buffer (Bio-Rad, UK) containing 2-mercaptoethanol the protein samples were loaded on 10% mini-PROTEANTGX Precast Gels (Bio-Rad) and separated by electrophoresis and then transferred onto PVDF membranes.

Immuno-detection used anti- DRP-2 primary antibody (Rb mAb to CRMP2 ab129082, abcam UK) and peroxidase linked secondary antibody (Goat pAb to Rb IgG (HRP) ab98467, abcam, UK). The blots were developed using the Opti4CN substrate kit (Bio-Rad) following the manufacturer’s recommended protocol and imaged using a Fujifilm LAS-3000 Imager. After imaging membranes were stripped using Restore™ Western Blot Stripping Buffer (Thermo Scientific) and re-probed using a primary antibody to beta-actin (Rb pAB to beta-actin ab8227, abcam UK). Blot images were analysed using Image-J [25]. For each blot lane DRP-2 bands were normalised to the respective beta-actin loading control band prior to semi-quantitative analysis where lysates from cells treated with BSA alone were considered as equivalent to 1 and those from cells treated with PA and OA scored accordingly (*n* = 4).

### Mitochondrial staining

N42 hypothalamic neurons were cultured on glass coverslips in 24 well plates to 70% confluency. Cells were challenged with either 50 μM BSA, 200 μM OA or 200 μM PA in serum-free medium for 6 h. Cells were then stained with 500 nM MitoTracker® Red CMXRos (Thermo Scientific, UK) for 45 min, washed and fixed using 4% paraformaldehyde for 20 min on ice. Cover slips were then mounted on slides using Vectashield with DAPI (Vector Laboratories, Burlingame, CA, USA). Images were captured using a Leica DMR microscope fitted with a QImaging QICAM FAST 1394 digital camera. Neuronal mitochondrial content was analysed using the ImageJ mitophagy macro [26]. The percentage of the cell area occupied by the mitochondria was used as a measure of cellular mitochondrial content.

### Statistical analysis

Body weight, food intake and IPGTT data are represented as mean ± SEM and were analysed using GenStat (GenStat, 10th Edition, VSN International Ltd., Oxford) by Student’s T tests. Mitochondria neuronal content was analysed using a one-way analysis of variance (ANOVA) followed by a Tukey multiple comparison test. *P* < 0.05 was considered statistically significant.

## Results

### Body weight and food intake

**Fig. 1:**
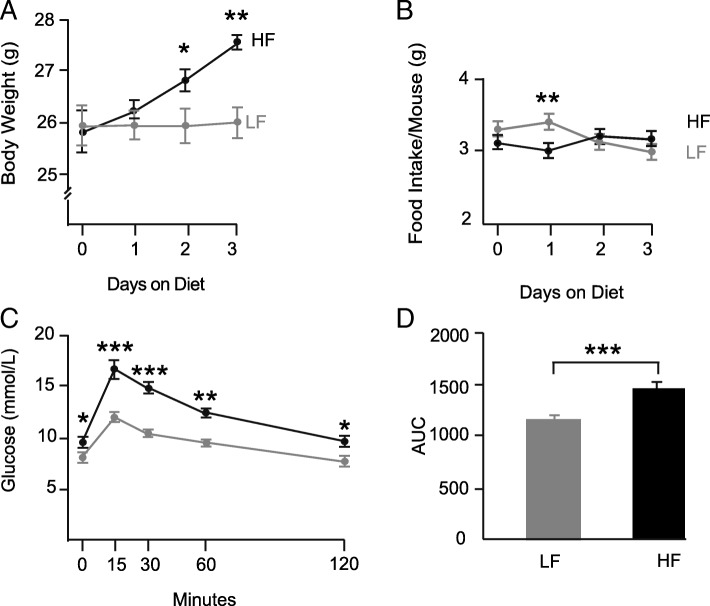
**a** Body weight of HFD mice was significantly higher than that of LFD mice after 2 and 3 days on diet **b** The intake of the HFD fed mice decreased after 1 day on diet but returned to LFD levels on days 2 and 3 (*n* = 6).**c** IPGTT in LFD and HFD fed mice after 3 days on diet. IPGTT was carried out as a non-recovery procedure as the effect of fasting and glucose administration can alter proteins for some time afterwards. Glucose levels at all time points tested was significantly higher in HFD fed mice (*n* = 8) (● HFD; ● LFD) as was AUC shown in (**d**). (* *P* < 0.05, **P < 0.01, ***P < 0.01)

The body weight of mice fed a HFD were significantly increased after 2 days (*P* < 0.05) and continued to increase up to 3 days on diet (*P* < 0.01) (Fig. 1a). Food intake (g) dropped significantly in HFD fed mice at day 1 (*P* < 0.01) but returned to LFD levels by 2 and 3 days of diet (Fig. 1b).

### Glucose tolerance

Basal glucose levels were higher in HFD fed mice after 3 days on diet (*P* < 0.05) and circulating glucose levels were higher at all time points tested after glucose challenge (IPGTT) (*P < 0.05, **P < 0.01, ***P < 0.001) as was the total AUC in HFD fed mice (*P* < 0.001) (Fig. 1c & d).

### Hypothalamic proteomics

**Fig. 2:**
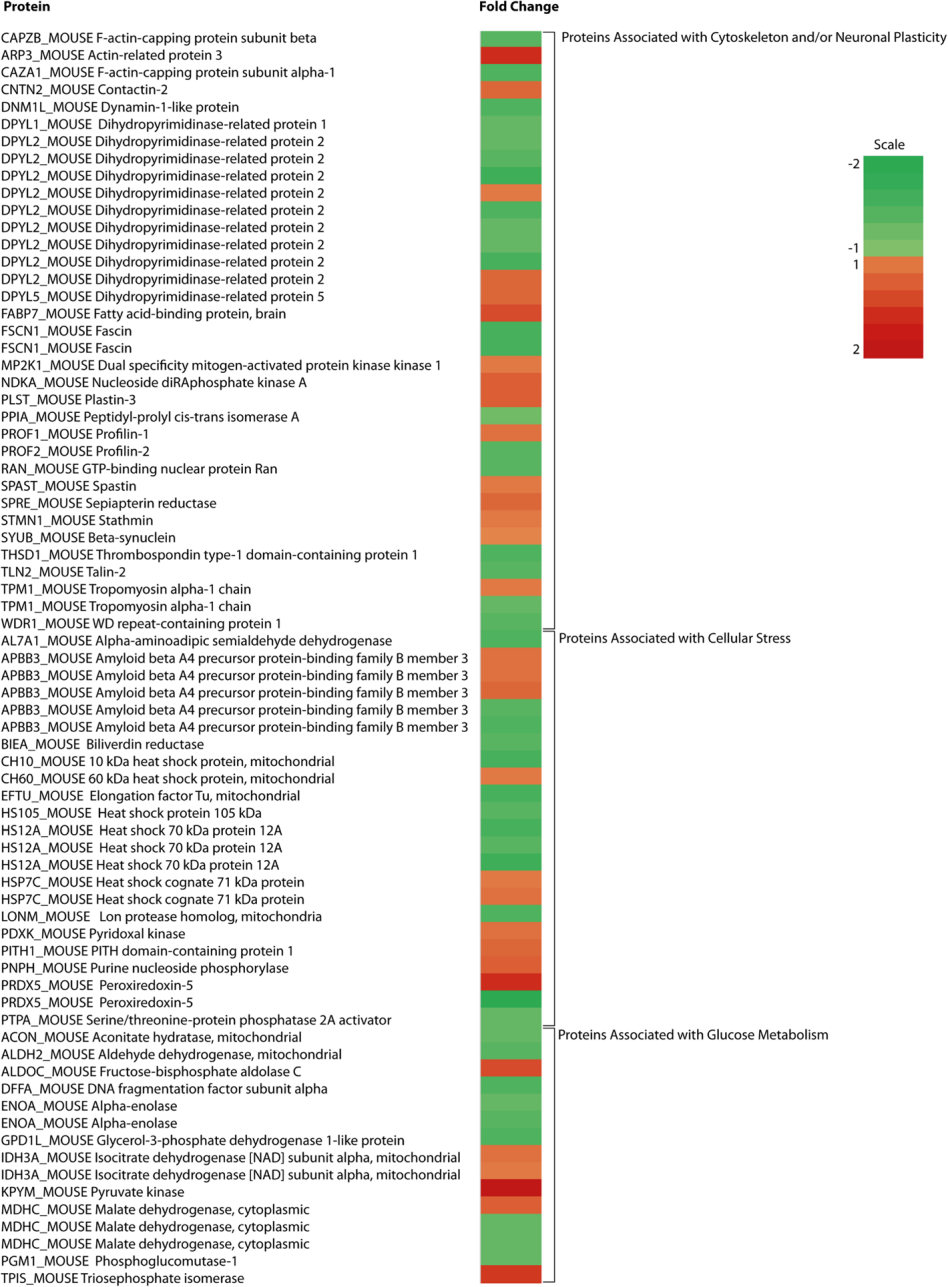
Heat map showing fold changes in proteins after 3 days of a HFD. Proteins are shown in multiples reflecting the number of spots which gave the same protein ID

2-DE analysis of mouse hypothalamic tissue revealed a total of 104 protein spots, from a total of 1147, that were significantly changed (*P* < 0.05) between the LFD and HFD fed mice after 3 days on diet (Additional file 2: Figure S1). These were further analysed by LC-MS/MS and identified as 78 unique proteins (Table 1 and Fig. 2).

**Table 1:** Protein identification by LC/MS/MS of spots in 2DE gels of 3 day mice hypothalamus which were significantly different in averaged normalised volume in HFD compared to LFD mice (*n* = 6). Proteins are arranged into 4 broad categories associated with specific functions: Proteins Associated with Cytoskeleton and/or Neuronal Plasticity, Proteins Associated with Cellular Stress, Proteins Associated with Energy Metabolism, all remaining proteins are grouped under Proteins Associated with Other Functions. Proteins belonging in more than one category are marked with an asterix (*) next to their UniProtKB identifier References for Table 1 can be found in Additional file 3

Spot #	Anova (p)	Fold Change	Protein Name	pI	MW	Average Normalised Volumes		Other Functional Categories	MASCOT Data	UniProtKB
						Low Fat	High Fat			
Proteins Associated with Cytoskeleton and/or Neuronal Plasticity										
24	8.14E-04	1.2	Dihydropyrimidinase-related protein 2	6.48	71,179	785,600	644,200	●Alzheimer’s disease/Neuronal degeneration (s1-s7)	Score: 56 Matches: 5(3) Sequences: 5(3)	O08553
33a	1.00E-03	1.3		6.46	67,358	431,700	342,400		Score: 56 Matches: 5(3) Sequences: 5(3)	
33b	8.00E-03	1.6		6.38	64,410	41,860	25,450		Score: 56 Matches: 5(3) Sequences: 5(3)	
22	2.00E-03	1.2		6.07	75,305	345,200	426,100		Score: 54 Matches: 5(2) Sequences: 5(2)	
34	4.00E-03	1.4		6.58	66,703	130,200	92,110		Score: 96 Matches: 6(2) Sequences: 6(2)	
27	7.00E-03	1.2		6.72	69,760	1,976,000	1,616,000		Score: 555 Matches: 68(37) Sequences: 17(13)	
90	9.00E-03	1.2		6.58	70,524	660,200	534,100		Score: 446 Matches: 50(25) Sequences: 16(14)	
25	1.90E-02	1.5		6.59	74,017	115,700	75,120		Score: 66 Matches: 5(2) Sequences: 5(2)	
23	3.70E-02	1.4		6.35	72,817	26,820	36,860		Score: 159 Matches: 17(10) Sequences:12(9)	
92	1.50E-02	1.2	Dihydropyrimidinase-related protein 1	7.27	67,795	342,400	274,800		Score: 104 Matches: 14(5) Sequences: 12(5)	P97427
35	3.50E-02	1.4	Dihydropyrimidinase-related protein 5	8.18	71,943	553,000	752,200	(s8)	Score: 44 Matches: 4(1) Sequences: 4(1) emPAI: 0.06	Q9EQF6
37	1.00E-03	1.5	Fascin	6.65	54,476	202,200	137,500	●Astrocyte specific ●Alzheimer’s disease/Neuronal degeneration (s9-s11)	Score: 51 Matches: 2(1) Sequences: 2(1)	Q61553
125	4.00E-03	1.5		6.64	56,114	66,550	44,030		Score: 51 Matches: 2(1) Sequences: 2(1)	
86	1.00E-03	1.4	Thrombospondin type-1 domain-containing protein 1	6.44	89,787	21,030	14,960	●Cellular stress ●Astrocyte specific ●Alzheimer’s disease/Neuronal degeneration (s13)	Score: 36 Matches: 3(1) Sequences: 1(1)	Q9JM61*
17	2.00E-03	1.4	Contactin-2	6.16	77,287	157,100	214,400	●Alzheimer’s disease/Neuronal degeneration (s14)	Score: 35 Matches: 1(1) Sequences: 1(1)	Q61330
88	3.00E-03	1.4	Dynamin-1-like protein	6.65	88,262	44,130	31,300	●Cellular stress ●Mitochondrial (s15,s16)	Score: 56 Matches: 6(4) Sequences: 6(4)	Q8K1M6*
87	1.90E-02	1.4		6.52	88,872	44,600	32,400		Score: 37 Matches: 2(1) Sequences: 2(1)	
85	1.90E-02	1.3		7.21	91,921	31,110	24,030		Score: 53 Matches: 4(2) Sequences: 4(2)	
52	4.00E-03	1.7	F-actin-capping protein subunit alpha-1	6.06	35,065	33,650	55,680	●Alzheimer’s disease/Neuronal degeneration (s17, s18)	Score: 82 Matches: 1(1) Sequences: 1(1) e	P47753
58	1.00E-02	1.2	F-actin-capping protein subunit β	6	29,413	633,600	780,600		Score: 80 Matches: 6(2) Sequences: 6(2)	P47757
124	4.00E-03	1.3	Fatty acid-binding protein, brain	5.69	12,708	329,600	413,500	●Cellular stress ●Astrocyte specific ●Alzheimer’s disease/Neuronal degeneration (s19-s21)	Score: 47 Matches: 2(1) Sequences: 2(1)	P51880*
99	4.00E-03	1.3	Dual specificity mitogen-activated protein kinase kinase 1	6.78	45,886	588,200	453,000	●Alzheimer’s disease/Neuronal degeneration (s22-s24)	Score: 100 Matches: 7(4) Sequences: 6(4)	P31938
82	5.00E-03	2	Profilin-1	9.98	12,625	23,210	47,490	●Alzheimer’s disease/Neuronal degeneration (s25-s28)	Score: 73 Matches: 5(2) Sequences: 3(2)	P62962
122	7.00E-03	1.5	Profilin-2	5.65	13,875	115,800	171,500		Score: 62 Matches: 2(2) Sequences: 2(2)	Q9JJV2
96	8.00E-03	1.5	Actin-related protein 3	6.18	50,983	31,780	47,350	●Alzheimer’s disease/Neuronal degeneration (s29)	Score: 47 Matches: 4(1) Sequences: 4(1)	Q99JY9
93	1.20E-02	1.5	Plastin-3	6.09	68,341	25,000	36,730	●Alzheimer’s disease/Neuronal degeneration (s30)	Score: 65 Matches: 5(3) Sequences: 5(3)	Q99K51
76	1.50E-02	1.4	Nucleoside diRAphosphate kinase A	7.14	15,333	279,200	205,400	●Alzheimer’s disease/Neuronal degeneration (s31, s32)	Score: 101 Matches: 15(8) Sequences: 6(4) emPAI: 1.23	P15532
116	1.70E-02	1.3	GTP-binding nuclear protein Ran	7.61	24,820	524,100	398,300	●Alzheimer’s disease/Neuronal degeneration (s33, s34)	Score: 131 Matches: 11(5) Sequences: 6(4)	P62827
26	2.10E-02	1.3	WD repeat-containing protein 1	6.89	73,690	146,600	116,800	(s35, s36)	W Score: 65 Matches: 11(3) Sequences: 8(3)	O88342
55	2.30E-02	1.2	T Tropomyosin alpha-1 chain	4.72	30,497	340,400	407,500	●Alzheimer’s disease/Neuronal degeneration (s37, s38)	Score: 70 Matches: 3(3) Sequences: 3(3)	P58771
89	2.10E-02	1.2		6.47	87,348	30,950	25,160		Score: 119 Matches: 9(3) Sequences: 9(3)	
69	2.60E-02	1.2	Stathmin	6.07	16,606	642,900	761,200	●Alzheimer’s disease/Neuronal degeneration (s39, s40)	Score: 36 Matches: 2(1) Sequences: 2(1)	P54227
60	2.60E-02	1.3	Talin-2	6.66	31,968	63,640	47,710	(s41)	Score: 34 Matches: 2(1) Sequences: 2(1)	Q71LX4
63	2.70E-02	1.4	Sepiapterin reductase	6.05	26,394	111,900	157,000	●Alzheimer’s disease/Neuronal degeneration (s42)	Score: 34 Matches: 2(1) Sequences: 2(1)	Q64105
74	3.10E-02	1.2	Spastin	4.91	15,061	311,900	375,100	●Alzheimer’s disease/Neuronal degeneration (s43-s45)	Score: 34 Matches: 1(1) Sequences: 1(1)	Q9QYY8
71	4.80E-02	1.1	Peptidyl-prolyl cis-trans isomerase A	7.33	16,394	173,900	153,400	●Alzheimer’s disease/Neuronal degeneration (s46-s48)	Score: 53 Matches: 4(2) Sequences: 4(2)	P17742
67	3.80E-02	1.1	Beta-synuclein	4.13	17,576	1,879,000	2,051,000	●Alzheimer’s disease/Neuronal degeneration (s49, s50)	Score: 57 Matches: 2(1) Sequences: 1(1)	Q91ZZ3
Proteins Associated with Cellular Stress										
20a	1.00E-03	1.6	Heat shock 70 kDa protein 12A	6.68	79,878	61,440	39,140	●Alzheimer’s disease/Neuronal degeneration (s51, s52)	Score: 69 Matches: 4(2) Sequences: 4(2)	Q8K0U4
20b	3.00E-03	1.5		6.71	83,079	95,300	64,690		Score: 69 Matches: 4(2) Sequences: 4(2)	
20c	1.50E-02	1.3		6.75	80,640	119,400	88,760		Score: 69 Matches: 4(2) Sequences: 4(2)	
79	8.00E-03	1.5	10 kDa heat shock protein, mitochondrial	7.29	10,958	645,800	427,900	●Mitochondrial (s53)	Score: 47 Matches: 3(1) Sequences: 3(1)	Q64433
31	2.50E-02	1.2	60 kDa heat shock protein, mitochondrial	5.79	63,646	1,008,000	1,245,000	●Mitochondrial ●Alzheimer’s disease/Neuronal degeneration (s54)	Score: 604 Matches: 70(41) Sequences: 18(15)	P63038
21	2.90E-02	1.2	Heat shock cognate 71 kDa protein	5.73	75,915	4,300,000	5,025,000	●Alzheimer’s disease/Neuronal degeneration (s2)	Score: 51 Matches: 7(2) Sequences: 7(2)	P63017
42	2.90E-02	1.3		8.07	48,958	1,434,000	1,799,000		Score: 183 Matches: 10(7) Sequences: 8(6)	
19	9.00E-03	1.3	Heat shock protein 105 kDa	6.72	88,567	67,300	50,100	(s55)	Score: 35 Matches: 1(1) Sequences: 1(1)	Q61699
114	1.00E-03	1.4	PITH domain-containing protein 1	5.95	25,039	52,050	73,410		Score: 66 Matches: 6(3) Sequences: 6(3)	Q8BWR2
45	2.00E-03	1.5	Elongation factor Tu, mitochondrial	7	46,325	170,000	110,000	●Mitochondrial ●Alzheimer’s disease/Neuronal degeneration s56)	Score: 66 Matches: 6(4) Sequences: 6(4)	Q8BFR5
165	2.00E-03	1.3	Amyloid beta A4 precursor protein-binding family B member 3	6.16	23,597	101,500	135,700	●Alzheimer’s disease/Neuronal degeneration (s57)	Score: 44 Matches: 7(1) Sequences: 1(1)	Q8R1C9
15	1.70E-02	1.3		6.05	88,110	31,970	40,700		Score: 51 Matches: 8(3) Sequences: 1(1)	
38	3.80E-02	1.4		7.25	55,131	165,400	114,700		Score: 49 Matches: 10(5) Sequences: 3(1)	
48	8.00E-03	1.3		6.83	41,004	128,000	95,720		Score: 55 Matches: 8(3) Sequences: 1(1)	
2	6.00E-03	1.4		5.91	211,272	47,200	66,770		Score: 48 Matches: 11(3) Sequences: 1(1)	
8	4.00E-03	1.4	Lon protease homolog, mitochondria	6.78	143,046	95,550	66,140	●Mitochondrial (s58)	Score: 50 Matches: 4(2) Sequences: 4(2)	Q8CGK3
112	5.00E-03	1.3	Biliverdin reductase	7.26	32,355	306,500	241,400	●Alzheimer’s disease/Neuronal degeneration (s59)	Score: 49 Matches: 5(2) Sequences: 5(2)	Q9CY64
106	6.00E-03	1.3	Pyridoxal kinase	6.12	37,439	541,300	681,500	●Alzheimer’s disease/Neuronal degeneration (s60, s61)	Score: 142 Matches: 11(8) Sequences: 8(7)	Q8K183
47	9.00E-03	1.2	Serine/threonine-protein phosphatase 2A activator	6.5	40,401	325,700	261,300	●Alzheimer’s disease/Neuronal degeneration (s62)	Score: 55 Matches: 4(1) Sequences: 4(1)	P58389
113	1.00E-02	1.5	Purine nucleoside phosphorylase	6.15	29,645	60,300	89,000	●Alzheimer’s disease/Neuronal degeneration (s63, s64)	Score: 56 Matches: 6(2) Sequences: 6(2) e	P23492
77	1.30E-02	2	Peroxiredoxin-5	8.69	15,091	208,000	409,900	●Mitochondrial ●Alzheimer’s disease/Neuronal degeneration (s65, s66)	Mass: 22226 Score: 181 Matches: 14(9) Sequences: 8(6)	P99029
78	3.20E-02	1.9		9.13	15,121	155,800	83,470		Score: 84 Matches: 9(4) Sequences: 6(4) emPAI: 0.88	
94	1.70E-02	1.4	Alpha-aminoadipic semialdehyde dehydrogenase	6.57	59,170	56,390	41,730	(67)	Score: 110 Matches: 6(4) Sequences: 6(4)	Q9DBF1
49	4.00E-02	1.7	Fructose-bisphosphate aldolase C	8	40,291	67,960	114,800	●Glucose metabolism ●Mitochondrial ●Alzheimer’s disease/Neuronal degeneration (s68-s70)	Score: 143 Matches: 8(5) Sequences: 8(5)	P05063*
95	4.20E-02	1.3	Aldehyde dehydrogenase, mitochondrial	6.79	54,367	713,100	564,900	●Mitochondrial ●Alzheimer’s disease/Neuronal degeneration (s71, s72)	Score: 160 Matches: 14(10) Sequences: 11(9)	P47738
101	4.40E-02	1.4	DNA fragmentation factor subunit alpha	6.93	43,473	200,500	147,500	●Alzheimer’s disease/Neuronal degeneration(s73, 7 s4)	Score: 42 Matches: 1(1) Sequences: 1(1)	O54786
Proteins Associated with Energy Metabolism										
13	5.09E-04	1.2	Aconitate hydratase, mitochondrial	7.99	93,902	617,800	528,900	●Cellular stress ●Mitochondrial ●Alzheimer’s disease/Neuronal degeneration(s9, s75)	Score: 159 Matches: 16(6) Sequences: 13(6)	Q99KI0*
53	9.00E-03	1.5	Malate dehydrogenase, cytoplasmic	6.07	34,445	68,430	104,200	●Alzheimer’s disease/Neuronal degeneration (s9)	Score: 65 Matches: 4(1) Sequences: 4(1) emPAI: 0.11	P14152
108	1.50E-02	1.2		6.46	37,603	129,700	104,000		Score: 62 Matches: 2(1) Sequences: 2(1)	
109	7.00E-03	1.2		6.53	34,252	3,506,000	2,996,000		Score: 435 Matches: 39(24) Sequences: 10(7)	
104	7.00E-03	1.3	Isocitrate dehydrogenase [NAD] subunit alpha, mitochondrial	6.06	41,608	130,800	164,600	●Mitochondrial ●Alzheimer’s disease/Neuronal degeneration (s76, s77)	Score: 73 Matches: 6(3) Sequences: 6(3)	Q9D6R2
105	7.00E-03	1.2		6.04	40,456	647,600	774,800		Score: 205 Matches: 21(12) Sequences: 13(8)	
117	9.00E-03	1.9	Triosephosphate isomerase	8.56	26,200	94,250	178,000	●Alzheimer’s disease/Neuronal degeneration (s78, s79)	Score: 128 Matches: 13(7) Sequences: 6(5)	P17751
41	1.00E-02	1.2	Alpha-enolase	6.89	48,793	3,024,000	2,527,000	●Alzheimer’s disease/Neuronal degeneration (s75, s80)	Score: 310 Matches: 55(26) Sequences: 15(12)	P17182
40	1.70E-02	1.3		6.66	49,013	1,724,000	1,284,000		Score: 125 Matches: 17(9) Sequences: 11(7)	
97	1.20E-02	2.5	Pyruvate kinase	8.82	48,848	47,750	117,800	●Alzheimer’s disease/Neuronal degeneration (s81)	Score: 121 Matches: 14(8) Sequences: 10(7)	P52480
49	2.60E-02	1.2	Phosphoglucomutase-1	7.02	67,904	384,900	309,800	●Alzheimer’s disease/Neuronal degeneration (s82, s83)	Score: 349 Matches: 28(18) Sequences: 20(17)	Q9D0F9
110	2.70E-02	1.4	Glycerol-3-phosphate dehydrogenase 1-like protein	7	39,084	138,600	99,230	●Alzheimer’s disease/Neuronal degeneration (s84)	Score: 160 Matches: 12(7) Sequences: 11(7)	Q3ULJ0
49	4.00E-02	1.7	Fructose-bisphosphate aldolase C	8	40,291	67,960	114,800	●Cellular stress ●Mitochondrial ●Alzheimer’s disease/Neuronal degeneration (s68-s71)	Score: 143 Matches: 8(5) Sequences: 8(5)	P05063*
64	4.30E-02	1.2	Phosphoglycerate mutase 1	7.32	27,981	999,700	830,600	●Alzheimer’s disease/Neuronal degeneration(s85)	Score: 127 Matches: 12(5) Sequences: 6(4)	Q9DBJ1
Protein Associated with Other Functions										
107	5.94E-04	1.4	Glycine--tRNA ligase	6.47	81,250	112,900	79,050	Protein synthesis ●Alzheimer’s disease/Neuronal degeneration (s86)	Score: 82 Matches: 10(4) Sequences: 9(4)	Q9CZD3
98	8.10E-04	1.5	RNA polymerase II-associated factor 1 homolog	6.49	44,734	131,600	88,860	Regulation of transcription, Wnt signaling pathway ●Alzheimer’s disease/Neuronal degeneration (s87)	Score: 39 Matches: 2(1) Sequences: 2(1)	Q8K2T8
16	8.11E-04	1.4	Ski oncogene	6.15	88,567	83,770	115,100	Signalling, Inhibits TGF beta signalling	Score: 37 Matches: 3(1) Sequences: 1(1)	Q60698
9	1.70E-02	1.6		6.82	119,868	157,100	99,730		Score: 34 Matches: 1(1) Sequences: 1(1)	
36	4.00E-03	1.6	Histidine--tRNA ligase, cytoplasmic	6.16	55,349	88,260	144,700	Protein synthesis ●Alzheimer’s disease/Neuronal degeneration (s88)	Score: 136 Matches: 10(6) Sequences: 9(6)	Q61035
7a	4.00E-03	1.4	Putative helicase Mov10l1	6.14	123,179	29,440	42,400	Negative regulation of cell cycle	Score: 43 Matches: 1(1) Sequences: 1(1)	Q99MV5
7b	6.00E-03	1.5		6.19	121,854	32,970	49,230		Score: 43 Matches: 1(1) Sequences: 1(1)	
44	5.00E-03	1.3	Rab GDP dissociation inhibitor beta	6.65	47,312	412,900	311,300	Signalling, Positive regulation of GTPase activity ●Alzheimer’s disease/Neuronal degeneration (s89)	Score: 104 Matches: 9(4) Sequences: 8(4)	Q61598
84	6.00E-03	1.6	Transcription termination factor 1	6.12	99,390	8292.349	13,440	Regulation of transcription	Score: 36 Matches: 2(1) Sequences: 2(1)	Q62187
32	7.00E-03	1.5	HMG box-containing protein	6.12	64,083	137,800	200,000	Regulation of transcription, Wnt signaling	Score: 40 Matches: 1(1) Sequences: 1(1)	Q8R316
118	8.00E-03	1.4	UMP-CMP kinase	6.16	22,194	133,000	187,600	Pyrimidine biosynthesis	Score: 91 Matches: 6(4) Sequences: 6(4)	Q9DBP5
100	9.00E-03	1.4	Paraspeckle component 1	6.86	46,160	178,800	126,900	Control of transcription Circadian rhythms	Score: 37 Matches: 3(0) Sequences: 3(0)	Q8R326
43	1.07E-02	1.3	Ornithine aminotransferase, mitochondrial	6.42	46,949	495,800	385,200	Amino acid metabolism ●Mitochondrial	Score: 90 Matches: 7(5) Sequences: 7(5)	P29758
61	1.10E-02	1.2	Omega-amidase NIT2	6.88	30,652	104,200	87,480	Amino acid metabolism	Score: 45 Matches: 3(1) Sequences: 3(1)	Q9JHW2
121	1.30E-02	1.5	Cytidine deaminase	5.63	15,212	32,230	47,840	Pyrimidine metabolism, Negative regulation of cell growth	Score: 34 Matches: 1(1) Sequences: 1(1)	P56389
59	1.70E-02	1.3	Haloacid dehalogenase-like hydrolase domain-containing protein 2	6.16	30,652	216,800	280,000	Metabolism, Dephosphorylation	Score: 50 Matches: 5(2) Sequences: 2(1)	Q3UGR5
120	1.80E-02	1.4	Sec1 family domain-containing protein 1	4.66	13,958	79,910	109,000	Cell morphogenesis, protein transport	Score: 48 Matches: 3(1) Sequences: 1(1)	Q8BRF7
103	1.80E-02	1.3	Ras-like protein family member 10B	6.07	43,143	78,700	105,900	Signaling	Score: 39 Matches: 1(1) Sequences: 1(1)	Q5SSG5
5	1.90E-02	1.5	Neutral alpha-glucosidase AB	6.13	146,358	32,530	47,230	Glycoprotein syntheses, Glycan metabolism	Score: 40 Matches: 3(1) Sequences: 3(1)	Q8BHN3
119	2.00E-02	1.4	Acylamino-acid-releasing enzyme	6.99	19,848	113,700	83,510	Beta amyloid processes, Proteolysis ●Alzheimer’s disease/Neuronal degeneration (s90)	Score: 39 Matches: 5(1) Sequences: 2(1)	Q8R146
111	2.10E-02	1.2	Phosphatidylinositol transfer protein alpha isoform	6.68	33,206	394,200	338,000	Transport of PtdIns and phosphatidylcholine, Axonogenesis	Score: 51 Matches: 11(1) Sequences: 7(1)	P53810
83	2.20E-02	1.3	Solute carrier family 12 member 1	4.62	98,323	38,480	51,740	Regulation of ionic balance and cell volume	Score: 39 Matches: 3(1) Sequences: 3(1)	P55014
14	2.30E-02	1.2	RalBP1-associated Eps domain-containing protein 2	5.21	83,232	525,500	609,200	Growth factor signaling, Cell migration	Score: 36 Matches: 1(1) Sequences: 1(1)	Q80XA6
115	2.40E-02	1.3	Isopentenyl-diphosphate delta-isomerase 2	6.13	25,465	19,710	26,100	Cholesterol metabolism	Score: 39 Matches: 1(1) Sequences: 1(1)	Q8BFZ6
102	2.50E-02	1.2	Glutamine synthetase	7.25	45,228	1,248,000	1,016,000	Positive regulation of synaptic transmission, Cellular response to starvation ●Alzheimer’s disease/Neuronal degeneration (s91)	Score: 160 Matches: 32(9) Sequences: 10(7)	P15105
70	2.70E-02	1.2	Glycolipid transfer protein	7.25	20,036	113,000	92,910	Glycolipid transport, Glucoceramide transport	Score: 36 Matches: 2(1) Sequences: 2(1)	Q9JL62
123	3.20E-02	1.2	Cystatin-B	7.14	12,833	63,610	52,090	Protease inhibitor, Negative regulation of proteolysis ●Alzheimer’s disease/Neuronal degeneration (s92)	Mass: 11153 Score: 45 Matches: 1(1) Sequences: 1(1)	Q62426

### Protein analysis according to function

We divided the proteins identified according to function. The changed proteins were found to be mainly associated with the cytoskeleton and synaptic plasticity (37 spots corresponding to 25 proteins), cellular stress responses (32 spots corresponding to 22 proteins), glucose metabolism (14 spots corresponding to 10 proteins). In addition, 28 spots corresponding to 26 proteins did not belong to any of these three functional categories. There are 5 proteins which fall into more than one functional category and these are marked with an asterisk next to the UniProt identifier and the other functional category listed. Mitochondrial proteins and those associated with mitochondrial function (10 proteins) and 3 astrocyte specific proteins are also identified. Additionally, many of the proteins changed in the present study have also been associated with neurodegenerative diseases, particularly Alzheimer’s disease (49 proteins) (Table 1 and Fig. 2).

### Western blotting

**Fig. 3:**
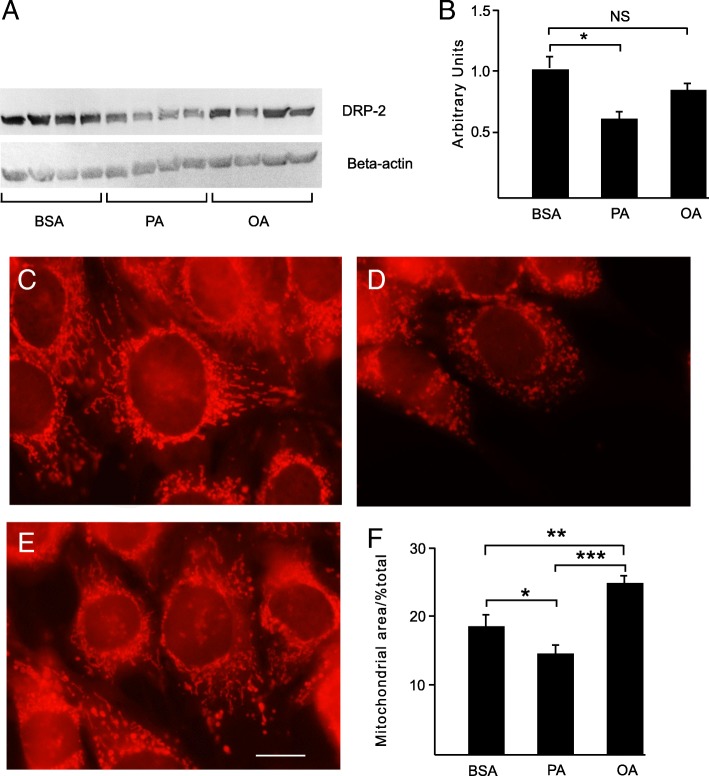
**a** Representative immunoblots showing changes in DRP-2 and beta-actin protein expression in response to fatty acid free BSA, PA and OA challenge in N42 cells **b** Quantification of DRP-2 bands normalised to beta-actin (*n* = 4 plates) BSA - bovine serum albumin, DRP-2 - dihydropyrimidinase-related protein 2, PA - palmitic acid, OA - oleic acid (* *P* < 0.05). **c**-**e** Representative fluorescence microscopy images of N42 hypothalamic neuronal mitochondria using MitoTracker® Red CMXRos. The red colour corresponds to mitochondria. Cells were challenged with, **c** fatty acid free BSA, **d** 200 μM PA and **e** 200 μM OA (Bar = 10 μm. Magnification = X100). **f** The percentage (%) area of the cell occupied by mitochondria after challenge. BSA - bovine serum albumin, PA - palmitic acid, OA - oleic acid (*P < 0.05, **P < 0.01, ***P < 0.001)

Immunoblotting of cell lysates from N42 hypothalamic neurons revealed that staining of the bands corresponding to DRP-2 protein was lower by ~ 38% (*P* < 0.05), in cells challenged with PA whereas those challenged with OA showed no significant changes in DRP-2 compared with control cells (Fig. 3a-b).

### Mitochondrial morphology and area occupied

Changes in mitochondrial shape were seen in cells challenged with PA but not after OA challenge. Mitochondria in PA challenged cells appeared rounded and isolated compared to control or OA challenged mitochondria which had an elongated and branched appearance (Fig. 3a-c). The percentage of the cell area occupied by mitochondria was significantly decreased when N42 hypothalamic neurons were challenged with PA (*P* < 0.05). The area occupied by mitochondria was increased when cells were challenged with OA relative to control cells (*P* < 0.01) (Fig. 3d).

## Discussion

C57Bl/6 J mice fed a semi-purified diet have been widely used in diet-induced obesity studies [20, 27] and in the present study HFD fed mice gained weight and developed glucose intolerance within 3 days as reported previously [20] confirming the reproducibility of the model and implying impaired hypothalamic function. Interestingly, in that study blood triglyceride levels were unchanged after 3 days on a HFD [20]. Evidence exists for the rapid, within 3 days, induction of hypothalamic insulin and leptin insensitivity [11, 28] by a HFD coupled with hypothalamic inflammation [8], endoplasmic reticulum (ER) stress [29, 30] and mitochondrial dysfunction [31].

In the present study, proteomic analysis of the hypothalamus confirmed the rapidity of HFD-induced changes and secondly demonstrated the large number of hypothalamic proteins (104 spots corresponding to 78 proteins) changed in response to a HFD. The validity of using a proteomics approach to interrogate hypothalamic changes is reinforced by the fact that as highly polarised cells, neurons, the major cell type present in the brain, are more likely to demonstrate translational modification of proteins at sites distant from the cell body to rapidly respond to stimuli rather than transcriptional changes and the subsequent transport of proteins from the cell body.

Unsurprisingly proteins involved in energy metabolism were altered in HFD. These include phosphoglucomutase-1 (PGM1), reported to sustain cell growth during nutritional changes by regulating the balance between glucose-1-phosphate and glucose-6-phosphate [32] and is differentially expressed in the brains of patients with Alzheimer’s disease [33]. A reduction of glucose utilisation is one of the earliest signs of Alzheimer’s disease with glucose metabolism adapting to oxidative stress by lowering levels of glycolysis and oxidative phosphorylation and increasing the generation of reducing factors such as nicotinamide adenine dinucleotide phosphate (NADPH) through the pentose phosphate pathway [34]. Two other enzymes altered by HFD are triosephosphate isomerase and phosphoglycerate mutase 1 both involved with the regulation of the glycolytic pathway. Mitochondrial aconitate hydratase, which catalyses the conversion of citrate to isocitrate in the tricarboxylic acid cycle showed the most significant change in HFD fed mice. It is linked to Alzheimer’s disease demonstrating lower activity in response to oxidative stress [34, 35] and loss of function due to oxidative damage in aging rat brain [36]. Isocitrate dehydrogenase which showed changes in two spots in HFD fed mice is also down-regulated in Alzheimer’s disease [37].Changes in these enzymes in response to HFD point to adaptations in metabolic pathways to overcome oxidative stress similar to those observed in the early stages of Alzheimer’s disease.

Glucose metabolism in the hypothalamus is likely impacted by the increase in circulating glucose seen on a HFD after 3 days. The entry of glucose into the brain is mediated by the non-insulin dependent glucose transporter, GLUT1 with brain glucose levels rising in parallel to circulating glucose concentrations. Excess glucose is neurotoxic via the polyol pathway, changing intracellular tonicity and increasing toxic AGEs which in combination with a HFD promote microglial reactivity [17].

Other protein changes are in pathways not previously thought to be part of the hypothalamic response to a HFD. These include 25 proteins associated with neurogenesis, synaptogenesis, neurite outgrowth and axonal and dendritic cytoskeletal proteins, implying that neuronal remodelling and changes in synaptic connectivity are changed and may be compromised. Notable amongst these are the collapsin response mediator family of proteins (CRMPS - also known as dihydropyrimidinase-related proteins - DPYL and DRPs), consisting of five closely sequence related, phosphoproteins. Single spots representing DRP-1 and 5 are changed together with 9 separate spots corresponding to DRP-2 demonstrating a large number of post-translational changes induced by a HFD. DRP-2 regulates microtubule dynamics and promotes the differentiation of axons from neurites by binding to tubulin dimers. This promotes microtubule assembly and stability [38] which in turn promotes axon elongation. Phosphorylation of DRP-2 lowers its binding affinity to tubulin and is key in the regulation of dendritic spine formation [39]. Because of this DRP-2 was selected to further study the effect of fatty acids in N42 hypothalamic cells where PA challenge altered expression of DRP-2 immunoreactive bands while OA had no effect supporting the contention that long-chain saturated fatty acids damage hypothalamic neuronal function [40].

Dendritic spines are small, highly dynamic, protrusions on the surface of dendrites, which form the postsynaptic component of excitatory synapses [41] and their formation in the hypothalamus is necessary for the activation of agouti-related peptide (AgRP) neurons by fasting [42]. Formation is dependent on cytoskeletal remodelling of actin [43] the most prominent cytoskeletal protein at synapses. Indeed a large number of proteins identified as changed by a HFD are associated with actin organisation, including F-actin-capping protein subunits alpha 1 and beta, profilin-1, profilin-2, plastin-3 and tropomyosin alpha-1 chain. Profilin-1 and 2 bind actin at synapses where they act both as stable structural components and as regulators of actin filament branching providing a modulatory component for the efficacy of pre- and post-synaptic terminals with actin being most enriched in dendritic spines [44, 45]. Also changed on a HFD was actin related protein 3 which functions as ATP-binding component of the Arp2/3 complex involved in regulation of actin polymerization important in dendritic spine formation [46]. Fascin appears twice on the list of proteins changed by a HFD and is important in the cross-linking of filamentous actin into ordered bundles present in cytoskeletal processes and in the function and architecture of cell protrusions again indicating changes in neuronal plasticity in response to a HFD.

Ornithine aminotransferase is also changed, and in the brain is involved in the synthesis of glutamate and gamma-aminobutyric acid GABA [47], two important neurotransmitters localised to synaptosomes [48], again indicating that communication between neurons is altered by a HFD.

Thus, a HFD has a rapid and profound effect on the hypothalamus, altering proteins involved in glucose metabolism linked to oxidative stress and other stress-related proteins. Unexpectedly a large number of cytoskeletal proteins involved in neuronal remodeling and synaptic plasticity were also changed indicating that this area of the brain was undergoing rapid structural changes in response to a HFD. Previously, it has been shown that in rodents susceptible to a HFD that synapses were lost from pro-opiomelanocortin (POMC) neurons which became sheathed in glia after 3 months on the diet [49].

Many of the proteins that were changed are also reported as altered in neurodegenerative diseases particularly Alzheimer’s disease (44 proteins). This may be due to the fact that both a HFD and Alzheimer’s disease are associated with neuro-inflammation and these changes are secondary to the pro-inflammatory condition or there may be a link between the neuronal effects of a HFD which leads to an Alzheimer’s type pathology as is borne out by the well documented connection between obesity, type 2 diabetes, cognitive decline and Alzheimer’s disease [50, 51]. Nonetheless, proteins which are associated with inflammation were not detected as changed by HFD in the present study and only 3 astrocyte specific proteins were identified.

The brain utilises high levels of energy compared to other organs and also contains elevated concentrations of lipids which are susceptible to peroxidation by reactive oxygen species (ROS) produced as a by-product of oxidative metabolism [52]. HFD-induced obesity is associated with oxidative stress and mitochondrial dysfunction, which are linked to neurodegeneration [53]. The effect of the long-chain saturated fatty acid, PA but not the monounsaturated fatty acid, OA, on mitochondrial function in neuronal cells is shown by distinct changes in mitochondrial morphology and area which are potentially indicative of fragmentation and loss of functionality.

## Conclusions

In conclusion, changes to synaptic plasticity and neuronal function appear to precede HFD-induced inflammation in the hypothalamus. Indeed at 3 days on a HFD no changes in any protein specifically related to inflammation were seen. Nonetheless, many proteins associated with cellular stress (22 proteins) were found to be changed in response to the diet indicating that oxidative stress in neurons may precede, and thus be, causative in hypothalamic inflammation. Further, experiments on N42 cells using the representative long-chain saturated fatty acid, PA, and the monounsaturated fatty acid, OA, confirm that the long-chain saturated fatty acids, rather than lipids per se, are causative in the changes seen with a HFD as shown by changes in mitochondrial morphology and immunoreactive DRP-2 levels.

## Additional files

Additional file 1: **Table S1.** Composition of the semi-purified diets used in the study (DOCX 15 kb)

Additional file 2: **Figure S1.** Representative 2D Coomassie stained gel of mouse hypothalamic proteins after 3 days on the HFD. Precision Blue Protein Standards (Bio-Rad) are shown as indicated. Numbered spots indicate those with significantly different average normalised volumes (*P* < 0.05) (*n* = 5) in HFD compared to (*P* < 0.06) LFD fed mice. Proteins were identified by LC/MS/MS. See Table 1 and Fig. 2 for protein identification (PDF 621 kb)

Additional file 3: Supplementary References (DOCX 27 kb)

## Abbreviations

2-DE: Two-dimensional gel electrophoresis
AgRP: Agouti-related peptide
ANOVA: Analysis of variance
AUC: Area under the curve
DRP-2: Dihydropyrimidinase-related protein 2
ER: Endoplasmic reticulum
GABA: Gamma-aminobutyric acid
GLUT1: Glucose transporter 1
HFD: High-fat diet
IKKβ/NKκB: Inhibitor of nuclear factor kappa-B kinase subunit β/nuclear factor kappa-light-chain-enhancer of activated B cells
IPG: Immobilized pH gradient
IPGTT: Intraperitoneal glucose tolerance test
LC − MS/MS: Liquid chromatography coupled with tandem mass spectrometry
LFD: Low-fat diet
N42: mHypoE-N42
Ob-Rb: Long signalling form of the leptin receptor
POMC: Pro-opiomelanocortin
ROS: Reactive oxygen species
TLR4: Toll like receptor 4
