# Supplementary material for: A high-fat diet induces rapid changes in the mouse hypothalamic proteome
Source: Nutr Metab (Lond). 2019 Apr 29;16:26. doi: 10.1186/s12986-019-0352-9 (PMC6489262; doi:10.1186/s12986-019-0352-9)
Supplement: Supplementary file 1 — Table S1. Composition of the semi-purified diets used in the study (DOCX 15 kb) [file 12986_2019_352_MOESM1_ESM.docx]

**Supplementary Table 1**

|  | **Control LFD**  **D12450B** | **HFD**  **D12492** |
| --- | --- | --- |
| **Composition** | **Kcal%** | |
| Protein | 20 | 20 |
| Carbohydrate | 70 | 200 |
| Fat | 10 | 60 |
| **Ingredients** | **Kcal** | |
| Casein | 800 | 800 |
| L-Cystine | 12 | 12 |
|  |  |  |
| Corn starch | 1260 | 0 |
| Maltodextrin | 140 | 125 |
| Sucrose | 1400 | 275.2 |
|  |  |  |
| Cellulose | 0 | 0 |
|  |  |  |
| Soybean oil | 225 | 225 |
| Lard | 180 | 2205 |
|  |  |  |
| Mineral Mix | 0 | 0 |
| Vitamin Mix | 40 | 40 |
|  |  |  |
| Dye | 0 | 0 |
