# Supplementary figures and images for: A high-fat diet induces rapid changes in the mouse hypothalamic proteome
Source: Nutr Metab (Lond). 2019 Apr 29;16:26. doi: 10.1186/s12986-019-0352-9 (PMC6489262; doi:10.1186/s12986-019-0352-9)

3

10

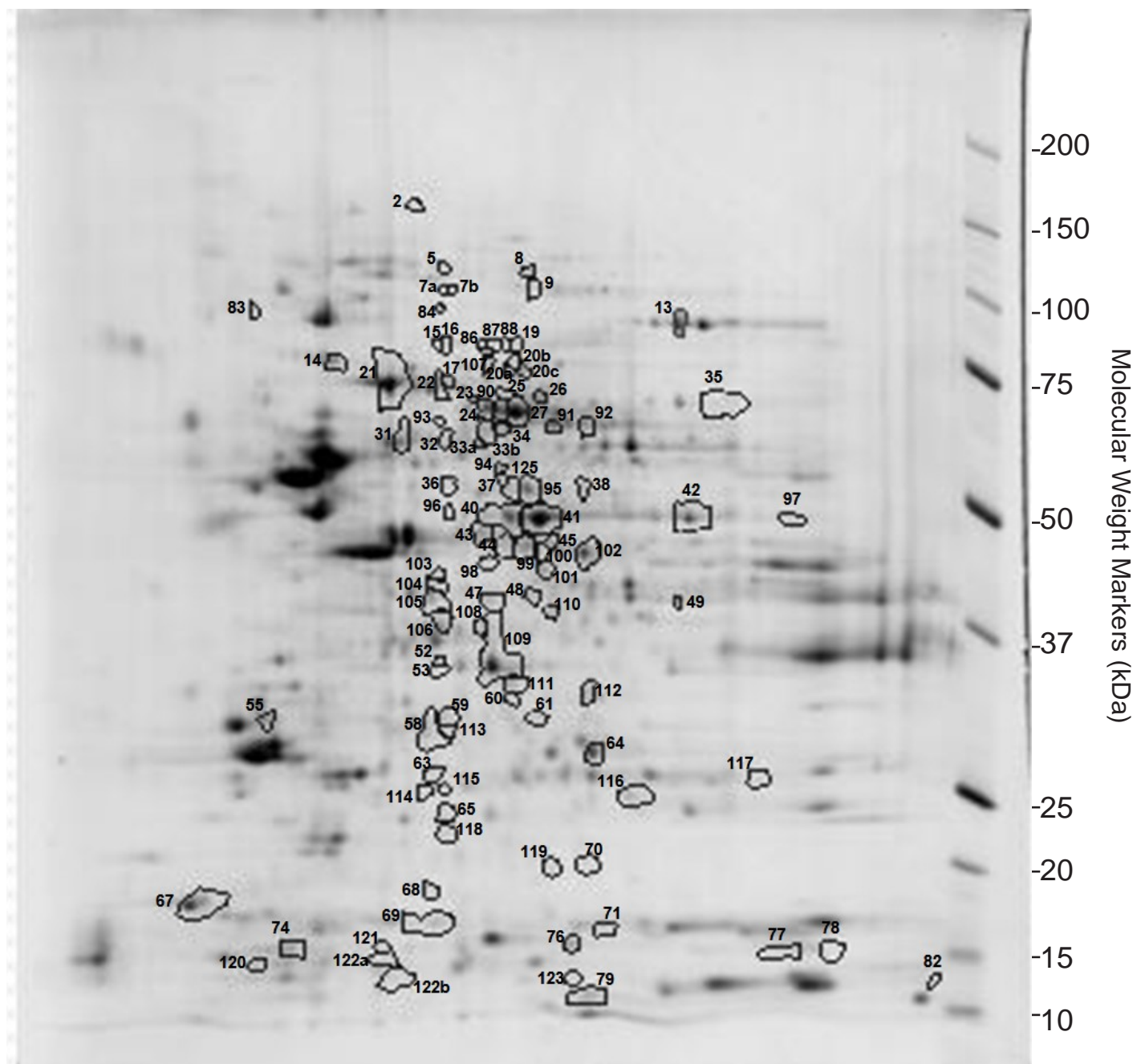

Supplement: Supplementary file 2 — Figure S1. Representative 2D Coomassie stained gel of mouse hypothalamic proteins after 3 days on the HFD. Precision Blue Protein Standards (Bio-Rad) are shown as indicated. Numbered spots indicate those with significantly different average normalised volumes (P < 0.05) (n = 5) in HFD compared to (P < 0.06) LFD fed mice. Proteins were identified by LC/MS/MS. See Table 1 and Fig. 2 for protein identification (PDF 621 kb) [file 12986_2019_352_MOESM2_ESM.pdf]
