# Supplementary material for: A high-fat diet induces rapid changes in the mouse hypothalamic proteome
Source: Nutr Metab (Lond). 2019 Apr 29;16:26. doi: 10.1186/s12986-019-0352-9 (PMC6489262; doi:10.1186/s12986-019-0352-9)
Supplement: Supplementary file 3 — Supplementary References (DOCX 27 kb) [file 12986_2019_352_MOESM3_ESM.docx]

**Supplementary References**

s1. Yang Y, Zhao B, Ji Z, Zhang G, Zhang J, Li S, et al. CRMPs colocalize and interact with cytoskeleton in hippocampal neurons. Int J Clin Exp Med. 2015;8:22337-44.

s2. Castegna A, Aksenov M, Thongboonkerd V, Klein JB, Pierce WM, Booze R, et al. Proteomic identification of oxidatively modified proteins in Alzheimer's disease brain. Part II: dihydropyrimidinase‐related protein 2, α‐enolase and heat shock cognate 71. J Neurochem. 2002;82:1524-32.

s3. Fu Y, Zhao D, Pan B, Wang J, Cui Y, Shi F, et al. Proteomic analysis of protein expression throughout disease progression in a mouse model of Alzheimer’s disease. J Alzheimer's Dis. 2015;47:915-26.

s4. Yamashita N, Goshima Y. Collapsin response mediator proteins regulate neuronal development and plasticity by switching their phosphorylation status. Mol Neurobiol. 2012;45:234-46.

s5 Hooli B, Kovacs-Vajna ZM, Mullin K, Blumenthal M, Mattheisen M, Zhang C, et al. Rare autosomal copy number variations in early-onset familial Alzheimer’s disease. Mol Psychiatry. 2014;19:676-81.

s6. Uchida Y, Ohshima T, Sasaki Y, Suzuki H, Yanai S, Yamashita N, et al. Semaphorin3A signalling is mediated via sequential Cdk5 and GSK3β phosphorylation of CRMP2: implication of common phosphorylating mechanism underlying axon guidance and Alzheimer's disease. Genes to Cells. 2005;10:165-79.

s7 Bretin S, Reibel S, Charrier E, Maus‐Moatti M, Auvergnon N, Thevenoux A, et al. Differential expression of CRMP1, CRMP2A, CRMP2B, and CRMP5 in axons or dendrites of distinct neurons in the mouse brain. J Comp Neurol. 2005;486:1-17.

s8. Brot S, Auger C, Bentata R, Rogemond V, Menigoz S, Chounlamountri N, et al. Collapsin response mediator protein 5 (CRMP5) induces mitophagy, thereby regulating mitochondrion numbers in dendrites. J Biol Chem. 2014;289:2261-76.

s9. Zahid S, Oellerich M, Asif AR, Ahmed N. Differential expression of proteins in brain regions of Alzheimer’s disease patients. Neurochem Res. 2014;39(1):208-15.

s10. Wei Z, Sun M, Liu X, Zhang J, Jin Y. Rufy3, a protein specifically expressed in neurons, interacts with actin‐bundling protein Fascin to control the growth of axons. J Neurochem. 2014;130:678-92.

s11. Castaño EM, Maarouf CL, Wu T, Leal MC, Whiteside CM, Lue L, et al. Alzheimer disease periventricular white matter lesions exhibit specific proteomic profile alterations. Neurochem Int. 2013;62:145-56.

s12. Ko C, Chu Y, Narumiya S, Chi J, Furuyashiki T, Aoki T, et al. The CCAAT/enhancer-binding protein delta/miR135a/thrombospondin 1 axis mediates PGE2-induced angiogenesis in Alzheimer's disease. Neurobiol Aging. 2015;36:1356-68.

s13. Son SM, Nam DW, Cha M, Kim KH, Byun J, Ryu H, et al. Thrombospondin-1 prevents amyloid beta–mediated synaptic pathology in Alzheimer's disease. Neurobiol Aging. 2015;36:3214-27.

s14. Masuda T. Contactin-2/TAG-1, active on the front line for three decades. Cell Adhesion & Migration. 2017:10:52.

s15. Uo T, Dworzak J, Kinoshita C, Inman DM, Kinoshita Y, Horner PJ, et al. Drp1 levels constitutively regulate mitochondrial dynamics and cell survival in cortical neurons. Exp Neurol. 2009;218:274-85.

s16. Fields JA, Serger E, Campos S, Divakaruni AS, Kim C, Smith K, et al. HIV alters neuronal mitochondrial fission/fusion in the brain during HIV-associated neurocognitive disorders. Neurobiol Dis. 2016;86:154-69.

s17. Kao PF, Banigan MG, Vanderburg CR, McKee AC, Polgar PR, Seshadri S, et al. Increased expression of TrkB and Capzb2 accompanies preserved cognitive status in early Alzheimer disease pathology. J Neuropathol Exp Neurol. 2012;71:654-64.

s18. Bartolini F, Ramalingam N, Gundersen GG. Actin-capping protein promotes microtubule stability by antagonizing the actin activity of mDia1. Mol Biol Cell. 2012;23:4032-40.

s19. Zetterberg H. Applying fluid biomarkers to Alzheimer's disease. Am J Physiol Cell Physiol. 2017;313:C3-C10.

s20. Schnell A, Chappuis S, Schmutz I, Brai E, Ripperger JA, Schaad O, et al. The nuclear receptor REV-ERBα regulates Fabp7 and modulates adult hippocampal neurogenesis. PloS one. 2014;9:e99883.

s21. Ebrahimi M, Yamamoto Y, Sharifi K, Kida H, Kagawa Y, Yasumoto Y, et al. Astrocyte‐expressed FABP7 regulates dendritic morphology and excitatory synaptic function of cortical neurons. Glia. 2016;64:48-62.

s22. Jeanneteau F, Deinhardt K, Miyoshi G, Bennett AM, Chao MV. The MAP kinase phosphatase MKP-1 regulates BDNF-induced axon branching. Nat Neurosci. 2010;13:1373-9.

s23 Li L, Mauric V, Zheng J, Kang SU, Patil S, Höger H, et al. Olfactory bulb proteins linked to olfactory memory in C57BL/6J mice. Amino Acids. 2010;39:871-86.

s24. Chen J, Zhou S, Zhang Y, Feng Y, Wang S. Glycosides of cistanche improve learning and memory in the rat model of vascular dementia. Eur Rev Med Pharmacol Sci. 2015;19:1234-40.

s25. Neuhoff H, Sassoè‐Pognetto M, Panzanelli P, Maas C, Witke W, Kneussel M. The actin‐binding protein profilin I is localized at synaptic sites in an activity‐regulated manner. Eur J Neurosci. 2005;21:15-25.

s26. Smith BN, Vance C, Scotter EL, Troakes C, Wong CH, Topp S, et al. Novel mutations support a role for Profilin 1 in the pathogenesis of ALS. Neurobiol Aging. 2015;36:1602. e17,

s27. Michaelsen-Preusse K, Zessin S, Grigoryan G, Scharkowski F, Feuge J, Remus A, et al. Neuronal profilins in health and disease: Relevance for spine plasticity and Fragile X syndrome. Proc Natl Acad Sci U S A. 2016;113:3365-70.

s28. Michaelsen K, Murk K, Zagrebelsky M, Dreznjak A, Jockusch BM, Rothkegel M, et al. Fine-tuning of neuronal architecture requires two profilin isoforms. Proc Natl Acad Sci U S A. 2010;107:15780-5.

s29. Ottis P, Loos M, Li KW, de Souza A, Schulz D, Smit AB, et al. Aging-Induced Proteostatic Changes in the Rat Hippocampus Identify ARP3, NEB2 and BRAG2 as a Molecular Circuitry for Cognitive Impairment. PloS one. 2013;8:e75112.

s30. Kaifer KA, Villalón E, Osman EY, Glascock JJ, Arnold LL, Cornelison D, et al. Plastin-3 extends survival and reduces severity in mouse models of spinal muscular atrophy. JCI insight. 2017;2:e89970

s31. Allard L, Burkhard PR, Lescuyer P, Burgess JA, Walter N, Hochstrasser DF, et al. PARK7 and nucleoside diphosphate kinase A as plasma markers for the early diagnosis of stroke. Clin Chem. 2005;51:2043-51.

s32. Boissan M, Montagnac G, Shen Q, Griparic L, Guitton J, Romao M, et al. Membrane trafficking. Nucleoside diphosphate kinases fuel dynamin superfamily proteins with GTP for membrane remodeling. Science. 2014;344:1510-5.

s33. Nagai M, Yoneda Y. Downregulation of the small GTPase Ras-related nuclear protein accelerates cellular ageing. Biochimica et Biophysica Acta (BBA)-General Subjects. 2013;1830:2813-9.

s34. Chen WS, Chen YJ, Huang YA, Hsieh BY, Chiu HC, Kao PY, et al. Ran-dependent TPX2 activation promotes acentrosomal microtubule nucleation in neurons. Sci Rep. 2017;7:42297.

s35. Ono S. Functions of actin-interacting protein 1 (AIP1)/WD repeat protein 1 (WDR1) in actin filament dynamics and cytoskeletal regulation. Biochem Biophys Res Commun. 2017;506:315-322

s36. Kuhns DB, Fink DL, Choi U, Sweeney C, Lau K, Priel DL, et al. Cytoskeletal abnormalities and neutrophil dysfunction in WDR1 deficiency. Blood. 2016;128:2135-43.

s37. Gormal R, Valmas N, Fath T, Meunier F. A role for tropomyosins in activity-dependent bulk endocytosis? Molecular and Cellular Neuroscience. 2017:84;112-118

s38. Brettle M, Patel S, Fath T. Tropomyosins in the healthy and diseased nervous system. Brain Res Bull. 2016;126:311-23.

s39. Shumyatsky GP, Malleret G, Shin R, Takizawa S, Tully K, Tsvetkov E, et al. Stathmin, a gene enriched in the amygdala, controls both learned and innate fear. Cell. 2005;123:697-709.

s40. Chauvin S, Sobel A. Neuronal stathmins: a family of phosphoproteins cooperating for neuronal development, plasticity and regeneration. Prog Neurobiol. 2015;126:1-18.

s41. Kerstein PC, Patel KM, Gomez TM. Calpain-Mediated Proteolysis of Talin and FAK Regulates Adhesion Dynamics Necessary for Axon Guidance. J Neurosci. 2017;37:1568-80.

s42. Takazawa C, Fujimoto K, Homma D, Sumi-Ichinose C, Nomura T, Ichinose H, et al. A brain-specific decrease of the tyrosine hydroxylase protein in sepiapterin reductase-null mice—as a mouse model for Parkinson’s disease. Biochem Biophys Res Commun. 2008;367:787-92.

s43. Zempel H, Luedtke J, Kumar Y, Biernat J, Dawson H, Mandelkow E, et al. Amyloid-beta oligomers induce synaptic damage via Tau-dependent microtubule severing by TTLL6 and spastin. EMBO J. 2013;32:2920-37.

s44. Matamoros AJ, Baas PW. Microtubules in health and degenerative disease of the nervous system. Brain Res Bull. 2016;126:217-25.

s45. Zempel H, Mandelkow E. Tau missorting and spastin-induced microtubule disruption in neurodegeneration: Alzheimer Disease and Hereditary Spastic Paraplegia. Molecular neurodegeneration. 2015;10:68.

s46 Pasetto L, Pozzi S, Castelnovo M, Basso M, Estevez AG, Fumagalli S, et al. Targeting Extracellular Cyclophilin A Reduces Neuroinflammation and Extends Survival in a Mouse Model of Amyotrophic Lateral Sclerosis. J Neurosci. 2017;37:1413-27.

s47 Halliday MR, Rege SV, Ma Q, Zhao Z, Miller CA, Winkler EA, et al. Accelerated pericyte degeneration and blood–brain barrier breakdown in apolipoprotein E4 carriers with Alzheimer’s disease. Journal of Cerebral Blood Flow & Metabolism. 2016;36:216-27.

s48. Arckens L, Van der Gucht E, Van den Bergh G, Massie A, Leysen I, Vandenbussche E, et al. Differential display implicates cyclophilin A in adult cortical plasticity. Eur J Neurosci. 2003;18:61-75.

s49. Cheng F, Vivacqua G, Yu S. The role of alpha-synuclein in neurotransmission and synaptic plasticity. J Chem Neuroanat. 2011;42:242-8.

s50. Surguchev AA, Surguchov A. Synucleins and Gene Expression: Ramblers in a Crowd or Cops Regulating Traffic? Frontiers in molecular neuroscience. 2017;10:224.

s51. N Fontaine S, D Martin M, A Dickey C. Neurodegeneration and the Heat Shock Protein 70 Machinery: Implications for Therapeutic Development. Current topics in medicinal chemistry. 2016;16:2741-52.

s52. Repalli J, Meruelo D. Screening strategies to identify HSP70 modulators to treat Alzheimer's disease. Drug Des Devel Ther. 2015;9:321-31.

s53. Owen JB, Opii WO, Ramassamy C, Pierce WM, Butterfield DA. Proteomic analysis of brain protein expression levels in NF-κβ p50−/− homozygous knockout mice. Brain Res. 2008;1240:22-30.

s54. Marino Gammazza A, Caruso Bavisotto C, Barone R, Macario ECd, JL Macario A. Alzheimer’s disease and molecular chaperones: current knowledge and the future of chaperonotherapy. Curr Pharm Des. 2016;22:4040-9.

s55. Hashikawa N, Utaka Y, Ogawa T, Tanoue R, Morita Y, Yamamoto S, et al. HSP105 prevents depression-like behavior by increasing hippocampal brain-derived neurotrophic factor levels in mice. Science Advances. 2017;3:e1603014.

s56. Boyd-Kimball D, Castegna A, Sultana R, Poon HF, Petroze R, Lynn BC, et al. Proteomic identification of proteins oxidized by Aβ (1–42) in synaptosomes: implications for Alzheimer's disease. Brain Res. 2005;1044:206-15.

s57. Csiszar A, Tucsek Z, Toth P, Sosnowska D, Gautam T, Koller A, et al. Synergistic effects of hypertension and aging on cognitive function and hippocampal expression of genes involved in beta-amyloid generation and Alzheimer's disease. Am J Physiol Heart Circ Physiol. 2013;305:H1120-30.

s58. Bota DA, Van Remmen H, Davies KJ. Modulation of Lon protease activity and aconitase turnover during aging and oxidative stress. FEBS Lett. 2002;532:103-6.

s59. Barone E, Di Domenico F, Cassano T, Arena A, Tramutola A, Lavecchia MA, et al. Impairment of biliverdin reductase-A promotes brain insulin resistance in Alzheimer disease: a new paradigm. Free Radical Biology and Medicine. 2016;91:127-42.

s60. Sorolla MA, Rodríguez-Colman MJ, Vall-Llaura N, Vived C, Fernández-Nogales M, Lucas JJ, et al. Impaired PLP-dependent metabolism in brain samples from Huntington disease patients and transgenic R6/1 mice. Metab Brain Dis. 2016;31:579-86.

s61. Vilariño‐Güell C, Wider C, Aasly JO, White LR, Rajput A, Rajput AH, et al. Association of pyridoxal kinase and Parkinson disease. Ann Neurol. 2010;67:409-11.

s62. Xiong Y, Liu F, Liu D, Huang H, Wei N, Tan L, et al. Opposite effects of two estrogen receptors on tau phosphorylation through disparate effects on the miR‐218/PTPA pathway. Aging cell. 2015;14:867-77.

s63. Peña-Altamira LE, Polazzi E, Giuliani P, Beraudi A, Massenzio F, Mengoni I, et al. Release of soluble and vesicular purine nucleoside phosphorylase from rat astrocytes and microglia induced by pro-inflammatory stimulation with extracellular ATP via P2X7 receptors. Neurochem Int. 2017.115:37-49

s64. Garcia‐Esparcia P, Hernández‐Ortega K, Ansoleaga B, Carmona M, Ferrer I. Purine metabolism gene deregulation in Parkinson's disease. Neuropathol Appl Neurobiol. 2015;41:926-40.

s65. Holley J, Newcombe J, Winyard P, Gutowski N. Peroxiredoxin V in multiple sclerosis lesions: predominant expression by astrocytes. Multiple Sclerosis Journal. 2007;13:955-61.

s66. Malty RH, Aoki H, Kumar A, Phanse S, Amin S, Zhang Q, et al. A map of human mitochondrial protein interactions linked to neurodegeneration reveals new mechanisms of redox homeostasis and NF-κB signaling. Cell systems. 2017;5:564-577

s67. Brocker C, Lassen N, Estey T, Pappa A, Cantore M, Orlova VV, et al. Aldehyde dehydrogenase 7A1 (ALDH7A1) is a novel enzyme involved in cellular defense against hyperosmotic stress. J Biol Chem. 2010;285:18452-63.

s68. Halford J, Shen S, Itamura K, Levine J, Chong AC, Czerwieniec G, et al. New astroglial injury-defined biomarkers for neurotrauma assessment. Journal of Cerebral Blood Flow & Metabolism. 2017;37:3278-99.

s69. Oppelt SA, Zhang W, Tolan DR. Specific regions of the brain are capable of fructose metabolism. Brain Res. 2017;1657:312-22.

s70. Chang RYK, Etheridge N, Dodd PR, Nouwens AS. Targeted quantitative analysis of synaptic proteins in Alzheimer’s disease brain. Neurochem Int. 2014;75:66-75.

s71. Chen C, Joshi AU, Mochly-Rosen D. The Role of Mitochondrial Aldehyde Dehydrogenase 2 (ALDH2) in Neuropathology and Neurodegeneration. Acta Neurologica Taiwanica. 2016;25:111-23.

s72. Li M, Zhang P, Wei H, Li M, Zou W, Li X, et al. Hydrogen sulfide ameliorates homocysteine-induced cognitive dysfunction by inhibition of reactive aldehydes involving upregulation of ALDH2. International Journal of Neuropsychopharmacology. 2016;20:305-15.

s73. Slane JM, Lee HS, Vorhees CV, Zhang J, Xu M. DNA fragmentation factor 45 deficient mice exhibit enhanced spatial learning and memory compared to wild-type control mice. Brain Res. 2000;867:70-9.

s74. Zhang J, Wang X, Bove KE, Xu M. DNA fragmentation factor 45-deficient cells are more resistant to apoptosis and exhibit different dying morphology than wild-type control cells. J Biol Chem. 1999;274:37450-4.

s75. Shen L, Chen C, Yang A, Chen Y, Liu Q, Ni J. Redox proteomics identification of specifically carbonylated proteins in the hippocampi of triple transgenic Alzheimer's disease mice at its earliest pathological stage. Journal of proteomics. 2015;123:101-13.

s76. Bubber P, Haroutunian V, Fisch G, Blass JP, Gibson GE. Mitochondrial abnormalities in Alzheimer brain: mechanistic implications. Ann Neurol. 2005;57:695-703.

s77. Naseri NN, Xu H, Bonica J, Vonsattel JPG, Cortes EP, Park LC, et al. Abnormalities in the tricarboxylic Acid cycle in Huntington disease and in a Huntington disease mouse model. Journal of Neuropathology & Experimental Neurology. 2015;74:527-37.

s78. Park S, Park HW, Kim N, Kim Y, Kwak M, Shin J, et al. Effects of Tau on the activity of triose phosphate isomerase (TPI) in brain cells. Neurochem Int. 2010;56:886-92.

s79. Tajes M, Eraso-Pichot A, Rubio-Moscardó F, Guivernau B, Ramos-Fernández E, Bosch-Morató M, et al. Methylglyoxal produced by amyloid-β peptide-induced nitrotyrosination of triosephosphate isomerase triggers neuronal death in Alzheimer's disease. J Alzheimer's Dis. 2014;41:273-88.

s80. Butterfield DA, Lange MLB. Multifunctional roles of enolase in Alzheimer’s disease brain: beyond altered glucose metabolism. J Neurochem. 2009;111:915-33.

s81. Harris RA, Tindale L, Cumming RC. Age-dependent metabolic dysregulation in cancer and Alzheimer’s disease. Biogerontology. 2014;15:559-77.

s82. Minjarez B, Calderón-González KG, Rustarazo MLV, Herrera-Aguirre ME, Labra-Barrios ML, Rincon-Limas DE, et al. Identification of proteins that are differentially expressed in brains with Alzheimer's disease using iTRAQ labeling and tandem mass spectrometry. Journal of proteomics. 2016;139:103-21.

s83. Ding B, Xi Y, Gao M, Li Z, Xu C, Fan S, et al. Gene expression profiles of entorhinal cortex in Alzheimer’s disease. American Journal of Alzheimer's Disease & Other Dementias®. 2014;29:526-32.

s84. El Kadmiri N, Slassi I, El Moutawakil B, Nadifi S, Tadevosyan A, Hachem A, et al. Glyceraldehyde-3-phosphate dehydrogenase (GAPDH) and Alzheimer's disease. Pathologie Biologie. 2014;62:333-6.

s85. Sultana R, Perluigi M, Newman SF, Pierce WM, Cini C, Coccia R, et al. Redox proteomic analysis of carbonylated brain proteins in mild cognitive impairment and early Alzheimer's disease. Antioxidants & redox signaling. 2010;12:327-36.

s86. Kapur M, Monaghan CE, Ackerman SL. Regulation of mRNA translation in neurons—a matter of life and death. Neuron. 2017;96:616-37.

s87. Zhang C, Mejia LA, Huang J, Valnegri P, Bennett EJ, Anckar J, et al. The X-linked intellectual disability protein PHF6 associates with the PAF1 complex and regulates neuronal migration in the mammalian brain. Neuron. 2013;78:986-93.

s88. Stum M, McLaughlin HM, Kleinbrink EL, Miers KE, Ackerman SL, Seburn KL, et al. An assessment of mechanisms underlying peripheral axonal degeneration caused by aminoacyl-tRNA synthetase mutations. Molecular and Cellular Neuroscience. 2011;46:432-43.

s89. Scheper W, Zwart R, Van der Sluijs P, Annaert W, Gool Wv, Baas F. Alzheimer’s presenilin 1 is a putative membrane receptor for rab GDP dissociation inhibitor. Hum Mol Genet. 2000;9:303-10.

s90. Yamin R, Bagchi S, Hildebrant R, Scaloni A, Widom RL, Abraham CR. Acyl peptide hydrolase, a serine proteinase isolated from conditioned medium of neuroblastoma cells, degrades the amyloid‐β peptide. J Neurochem. 2007;100:458-67.

s91 Kulijewicz-Nawrot M, Syková E, Chvátal A, Verkhratsky A, Rodríguez JJ. Astrocytes and glutamate homoeostasis in Alzheimer's disease: a decrease in glutamine synthetase, but not in glutamate transporter-1, in the prefrontal cortex. ASN neuro. 2013;5:AN20130017.

s92. Yang D, Stavrides P, Mohan PS, Kaushik S, Kumar A, Ohno M, et al. Reversal of autophagy dysfunction in the TgCRND8 mouse model of Alzheimer's disease ameliorates amyloid pathologies and memory deficits. Brain. 2010;134:258-77.
